# Selection of *Plasmodium falciparum pfcrt* and *pfmdr1* polymorphisms after treatment with artesunate–amodiaquine fixed dose combination or artemether–lumefantrine in Liberia

**DOI:** 10.1186/s12936-016-1503-3

**Published:** 2016-09-05

**Authors:** Sabina Dahlström Otienoburu, Oumou Maïga-Ascofaré, Birgit Schramm, Vincent Jullien, Joel J. Jones, Yah M. Zolia, Pascal Houzé, Elizabeth A. Ashley, Jean-René Kiechel, Philippe J. Guérin, Jacques Le Bras, Sandrine Houzé

**Affiliations:** 1 Institut de Médecine et d’Epidémiologie Appliquée, Bichat-C. Bernard Hospital, Paris, France; 2 WorldWide Antimalarial Resistance Network, Oxford, UK; 3 Johnson C. Smith University, Charlotte, NC 28216 USA; 4 Bernhard Nocht Institute for Tropical Medicine, 20359 Hamburg, Germany; 5 Epicentre, 75012 Paris, France; 6 INSERM U1129, Service de Pharmacologie, Hôpital Européen Georges Pompidou, Université Paris Descartes, 75015 Paris, France; 7 National Malaria Control Programme, Ministry of Health and Social Welfare, Monrovia, Liberia; 8 AP-HP, Saint-Louis Hospital Biochemistry Laboratory, Paris, France; 9 Nuffield Department of Clinical Medicine, Centre for Tropical Medicine and Global Health, University of Oxford, Oxford, UK; 10 Drugs for Neglected Diseases Initiative, Geneva, Switzerland; 11 IRD UMR216, Paris-Descartes University, Paris, France; 12 Parasitology Laboratory—French National Malaria Reference Centre, AP-HP, Bichat-C. Bernard Hospital, Paris, France

**Keywords:** *Plasmodium falciparum*, Malaria, Antimalarial agents, Artemisinin-based combination therapy, Drug resistance, Selection, *pfmdr1*, *pfcrt*, *pfmrp1*

## Abstract

**Background:**

*Plasmodium falciparum* uncomplicated malaria can successfully be treated with an artemisinin-based combination therapy (ACT). However resistance is spreading to the different ACT compounds; the artemisinin derivative and the partner drug. Studies of *P. falciparum* polymorphisms associated with drug resistance can provide a useful tool to track resistance and guide treatment policy as well as an in-depth understanding of the development and spread of resistance.

**Methods:**

The role of *P. falciparum* molecular markers in selection of reinfections was assessed in an efficacy trial comparing artesunate–amodiaquine fixed-dose combination with artemether–lumefantrine to treat malaria in Nimba County, Liberia 2008–2009. *P. falciparum* polymorphisms in *pfcrt* 76, *pfmdr1* 86, 184 and 1246, and *pfmrp1* 876 and 1466 were analysed by PCR-RFLP and pyrosequencing.

**Results:**

High baseline prevalence of *pfmdr1* 1246Y was found in Nimba county (38 %). *Pfmdr1* 1246Y and *pfmdr1* 86+184+1246 haplotypes NYY and YYY were selected in reinfections in the artesunate–amodiaquine arm and *pfcrt* K76, *pfmdr1* N86 and *pfmdr1* haplotype NFD were selected in artemether–lumefantrine reinfections. Parasites harbouring *pfmdr1* 1246Y could reinfect earlier after treatment with artesunate–amodiaquine and parasites carrying *pfmdr1* N86 could reinfect at higher lumefantrine concentrations in patients treated with artemether–lumefantrine.

**Conclusions:**

Although treatment is highly efficacious, selection of molecular markers in reinfections could indicate a decreased sensitivity or tolerance of parasites to the current treatments and the baseline prevalence of molecular markers should be closely monitored. Since individual drug levels and the day of reinfection were demonstrated to be key determinants for selection of reinfections, this data needs to be collected and taken into account for accurate evaluation of molecular markers for anti-malarial treatments.

The protocols for the clinical trial was registered with Current Controlled Trials, under the Identifier Number ISRCTN51688713 on 9 October 2008

## Background

*Plasmodium falciparum* malaria is a devastating disease still causing high mortality and morbidity especially in children in sub-Saharan Africa. The uncomplicated form of *P. falciparum* infection can be easily and successfully treated with an artemisinin-based combination therapy (ACT); however there is always the threat of resistance development to the different ACT compounds; the artemisinin derivative and/or the partner drug. Studies of *P. falciparum* polymorphisms associated with drug resistance can provide a useful tool to track resistance and guide treatment policy as well as an in-depth understanding of the development and spread of resistance.

Polymorphisms in *pfmdr1* and *pfcrt* have been shown to have an effect on parasite susceptibility to artesunate–amodiaquine (AS–AQ) treatment, in particular *pfcrt* 76T, *pfmdr1* 1246Y and the *pfmdr1* 86-184-1246 haplotype Y–Y–Y which has been associated with recrudescences and reinfections [1–4]. *Pfmdr1* N86, 184F and D1246 and *pfcrt* K76 alleles are repeatedly demonstrated to be selected in reinfections or recurrent infections after artemether–lumefantrine (AL) treatment [5, 6], supporting their role in the decreased sensitivity to lumefantrine. In pooled analyses, an increased risk of recrudescence after AL treatment was demonstrated when *pfmdr1* N86 was present [4].

Only scarce data on anti-malarial efficacy and prevalence of molecular resistance markers is available from Liberia. In 1978–1981, wildtype alleles *pfcrt* K76 and *pfmdr1*N86 were dominating [7]. In 2000, high clinical resistance to chloroquine was observed as well as high baseline prevalence of the chloroquine resistance marker *pfcrt* 76T (84 %) [8]. AS–AQ became the first line treatment in 2003 and it was changed to AS–AQ fixed dose combination (ASAQ-FDC) in 2010. High efficacy of ASAQ-FDC and AL was demonstrated in this clinical trial conducted in 2008–2009 [9]. The aim of the study was to investigate the prevalence and selection of *pfcrt* and *pfmdr1* genotypes in the clinical trial. This is the first study to assess molecular anti-malarial resistance markers in Liberia since the implementation of ACT. The work shows selection of parasite molecular markers in reinfections after treatment with both ACT and that molecular markers can influence the time after treatment and at which drug concentration a parasite is able to reinfect.

## Methods

### Study subjects

Analyses were performed of *P. falciparum* positive blood samples from a randomized non-inferiority efficacy trial evaluating ASAQ-FDC [n = 149 (ASAQ Winthrop®, Sanofi-Aventis)] and AL [n = 150 (Coartem®, Novartis)], conducted in 2008–2009 in Saclepea Comprehensive Health Centre in Nimba County, Liberia-a facility at the time supported by Médecins Sans Frontières-Switzerland in collaboration with the Ministry of Health. Children below 5 years of age were enrolled and followed for 42 days. The protocols for the clinical trial was registered with Current Controlled Trials, under the identifier number ISRCTN51688713 [9]. Blood was collected from the patients on FTA filter papers before treatment and during the follow up.

Parasite molecular markers in *pfcrt* K76T, *pfmdr1* N86Y, Y184F, D1246Y and *pfmrp1* I876V and K1466R was assessed in pre-treatment and post-treatment samples, classified according to the 42-day PCR-adjusted outcome. Recrudescences and reinfections were distinguished by stepwise genotyping *msp1*, *msp2* and *glurp* [9].

### DNA extraction

DNA was extracted from blood spotted onto FTA filter papers using the QIAamp® 96 DNA Blood Kit according to the manufacturer’s protocol for dried blood spots (Qiagen, Hilden, Germany).

### Pyrosequencing

*pfmrp1* SNPs in codons I876V and K1466R were analysed from extracted DNA by PCR amplification followed by pyrosequencing previously described [3]. The definition of a single genotype (pure) infection was a pyrosequencing result above 90 % or below 10 %, while a mixed genotype infection was between 10 and 90 % for both genotypes.

### Nested-PCR and RFLP

*Pfcrt*, and *pfmdr1* SNPs were analysed by nested PCR followed by restriction fragment length polymorphisms as previously described [3].

### Determination of drug concentrations in blood and serum

Pre-treatment serum samples were tested for previous intake of chloroquine and quinine by liquid chromatography coupled with tandem mass spectrometry. Pre-treatment and day 7 concentrations of desethylamodiaquine (DAQ) and lumefantrine were analysed from venous blood spotted on filter paper and measured by high performance liquid chromatography with ultraviolet detection (lumefantrine) or by tandem-mass spectrometry [4].

### Data analyses

Fisher’s two-tailed test (GraphPad Software Inc. Sand Diego, CA) was used to evaluate genetic selection by the difference in genotype prevalence between pre-treatment samples and recrudescences and reinfections, respectively. The baseline genotype prevalence was based on pre-treatment samples from both treatment arms. Patients were excluded from the pre-treatment group due to incorrect enrolment and infection with other species and from the post-treatment analysis due to infection with other species, missing or undetermined result from the *msp1, msp2* and *glurp* genotyping or incomplete treatment. In one SNP analysis, mixed genotypes were analysed together with the non-selected genotypes against the selected genotype, as previously suggested [3]. For example, in the AL arm the prevalence of mixed alleles in position *pfmdr1* 86 (N+Y) was added to the prevalence of *pfmdr1* 86Y and compared to the prevalence of *pfmdr1* N86. Mixed genotype infections were excluded in the haplotype analyses. Mann–Whitney U test was used to assess differences in drug concentrations at day 7 and time of reinfection in relation to parasite genotype. Exclusion criteria were incomplete treatment and missing data. Statistical significance was defined as a *p* value ≤0.05 in all analyses.

## Results

### Baseline prevalence of polymorphisms

Before treatment the prevalence of the *pfcrt* mutant allele 76T was 93.6 %. There was large variation in the *pfmdr1* SNPs where the mutant alleles 86Y, 184F and 1246Y were found in 69.4, 43.6 and 38.4 % respectively (including mixed genotype infections). The wild type allele was predominating in *pfmrp1*; I876 was found in 99.3 % of the samples and K1466R in 97.6 % (Table 1). Previous intake of chloroquine, quinine or amodiaquine/DAQ (measured in pre-treatment serum samples), did not have significant impact on the baseline prevalence of *pfcrt* and *pfmdr1* SNPs.

**Table 1:** Number and prevalence of *Plasmodium falciparum* polymorphisms pre- and post-treatment and post-treatment selection

Genotype	Pre-treatment		Post-treatment							
			ASAQ-FDC				AL			
			Recrudescences		Reinfections		Recrudescences		Reinfections	
*pfcrt* 76										
K	19	6.5 %	0	0.0 %	2	3.3 %	1	16.7 %	12***	*31.6* %
K/T	7	2.4 %	0	0.0 %	3	5.0 %	0	0.0 %	1	2.6 %
T	268	91.2 %	2	100.0 %	55	91.7 %	5	83.3 %	25	65.8 %
*pfmdr1* 86										
N	90	30.6 %	1	50.0 %	12	19.7 %	4	66.7 %	28***	*70.0* %
N/Y	47	16.0 %	0	0.0 %	8	13.1 %	1	16.7 %	3	7.5 %
Y	157	53.4 %	1	50.0 %	41	67.2 %	1	16.7 %	9	22.5 %
*pfmdr1* 184										
Y	166	56.5 %	1	50.0 %	33	55.9 %	3	50.0 %	24	55.8 %
Y/F	24	8.2 %	1	50.0 %	2	3.4 %	1	16.7 %	2	4.7 %
F	104	35.4 %	0	0.0 %	24	40.7 %	2	33.3 %	17	39.5 %
*pfmdr1* 1246										
D	181	61.6 %	2	66.7 %	29	48.3 %	5	83.3 %	33	76.7 %
D/Y	58	19.7 %	1	33.3 %	7	11.7 %	0	0.0 %	2	4.7 %
Y	55	18.7 %	0	0.0 %	24**	*40.0* %	1	16.7 %	8	18.6 %
*pfmrp1* 876										
I	285	96.9 %	2	100.0 %	59	96.7 %	6	100.0 %	42	97.7 %
I/V	7	2.4 %	0	0.0 %	1	1.6 %	0	0.0 %	0	0.0 %
V	2	0.7 %	0	0.0 %	1	1.6 %	0	0.0 %	1	2.3 %
*pfmrp1* 1466										
K	283	96.3 %	3	100.0 %	60	100.0 %	6	100.0 %	40	100.0 %
K/R	4	1.4 %	0	0.0 %	0	0.0 %	0	0.0 %	0	0.0 %
R	7	2.4 %	0	0.0 %	0	0.0 %	0	0.0 %	0	0.0 %
*pfmdr1 86* + *184* + *1246* ^*a*^										
NYD	55	27.8 %	1	100.0 %	5	10.2 %	2	40.0 %	15	42.9 %
NYY	0	0 %	0	0 %	2*	*4.1* %	0	0 %	1	2.9 %
NFD	29	14.6 %	0	0.0 %	4	8.2 %	2	40.0 %	10*	*28.6* %
YFD	54	27.3 %	0	0.0 %	15	30.6 %	0	0.0 %	1	2.9 %
YYY	47	23.7 %	0	0.0 %	20*	*40.8* %	1	20.0 %	5	14.3 %
Others^b^	13	6.6 %	0	0.0 %	3	6.1 %	0	0.0 %	3	8.6 %

Number and prevalence of polymorphisms selected post-treatment are in italics

* p ≤ 0.05, ** p < 0.001, *** p < 0.0001

^a^ Haplotype prevalence excluding mixed genotype infections

^b^ YYD, NFY, YFY

### Selection of polymorphisms after treatment

After treatment *pfmdr1* 1246Y was selected in the ASAQ-FDC arm, which was found in 55/294 (18.7 %) patients at baseline and in 24/60 (40.0 %) reinfections (Fisher, two-tailed, *p* < 0.001) (Table 1). In the AL arm, a selection of *pfcrt* K76 and *pfmdr1* N86 was identified, that increased in prevalence from 19/294 (6.5 %) and 90/294 (30.6 %) at baseline to 12/38 [31.6 % (*p* < 0.0001)] and 28/40 [70.0 % (*p* < 0.0001)], respectively, in reinfections (Table 1). These results were confirmed when omitting mixed genotype infections from the analyses. When studying the *pfmdr1* haplotype 86+184+1246, NYY and YYY were selected in reinfections after ASAQ-FDC treatment, with a baseline prevalence of 0/198 (0 %) and 47/198 (23.7 %) and reinfection prevalence of 2/49 [4.1 % (*p* = 0.04)] and 20/49 [40.8 % (*p* = 0.02)], respectively, while the prevalence of the wild-type haplotype NYD decreased from 27.8 to 10.2 % (*p* < 0.01). In AL reinfections the haplotype NFD was selected with a prevalence of 10/35 (28.6 %) compared to 29/198 (14.6 %) at baseline (*p* = 0.05), while the prevalence of the haplotype YFD significantly decreased from 27.3 % at baseline to 2.9 % (*p* < 0.001).

### Parasite genotypes in relation to day of reinfection and drug concentration

**Fig. 1:**
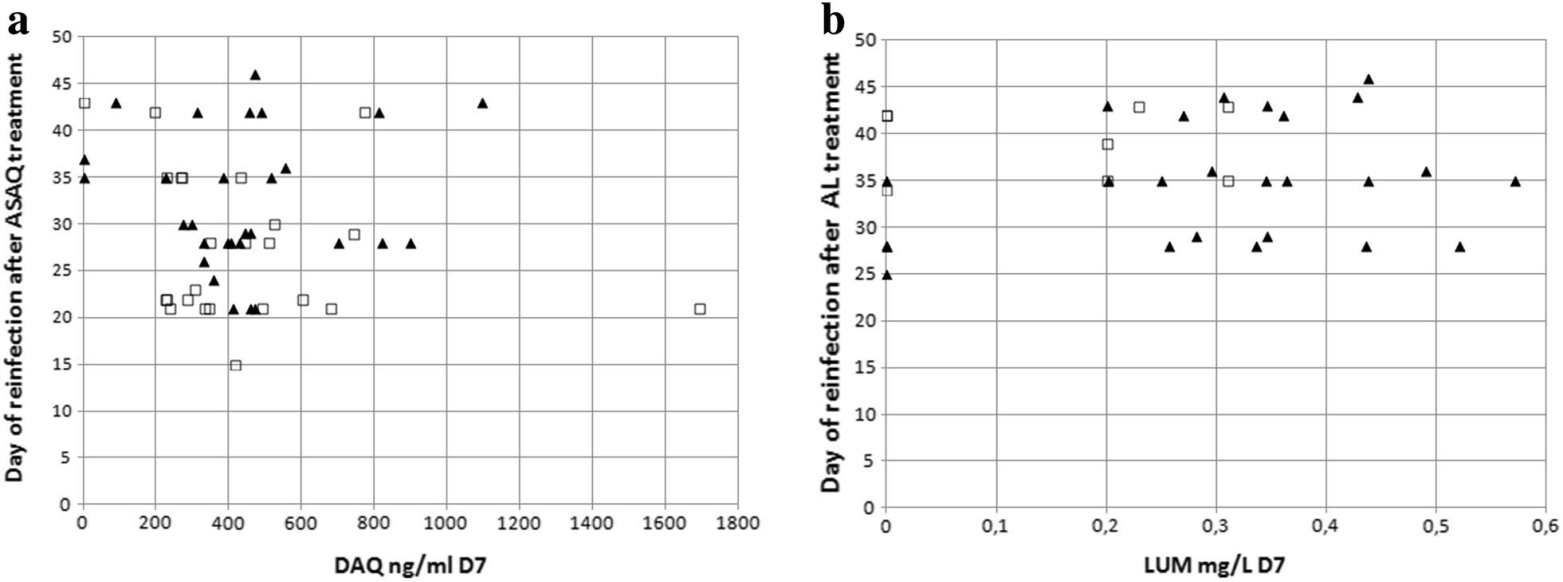
*Plot* of reinfection genotypes by day and patient blood drug concentration day 7. Each data point represents the *P. falciparum* genotype of a reinfection. **a** Genotype of reinfections after ASAQ-FDC treatment and desethylamodiaquine (DAQ) concentration. *Filled triangles* represent reinfections harbouring the *pfmdr1* D1246 genotype and *open squares* represent *pfmdr1* 1246Y reinfections. **b** Genotype of reinfections after AL treatment and lumefantrine (LUM) concentration. *Filled triangles* represent reinfections harbouring the *pfmdr1* N86 genotype and *open squares* represent *pfmdr1* 86Y reinfections

The correlation between single parasite genotypes and the time of reinfection or patient blood drug concentration was assessed. After ASAQ-FDC treatment, reinfecting parasites harbouring *pfmdr1* 1246Y were observed earlier (median, interquartile range; 25.5, 21–35 days) than reinfections with parasites carrying *pfmdr1* D1246 (30, 28–37 days, p = 0.03) (Fig. 1a). There was no significant difference in DAQ day 7 concentrations in patients infected with parasites with the respective genotypes. In patients treated with AL there was no significant difference in reinfection day between *pfmdr1* N86 and 86Y reinfections. Lumefantrine concentrations (day 7) were significantly higher in patients reinfected with parasites carrying *pfmdr1* N86 (median, interquartile range; 0.32, 0.21–0.41 mg/L) than in patients with *pfmdr1* 86Y reinfections (0.2, 0–0.24 mg/L, p = 0.03) (Fig. 1b). All reinfections that occurred in patients with day 7 lumefantrine concentrations higher than 0.31 mg/L were caused by parasites carrying *pfmdr1* N86. There were no significant differences associated with *pfcrt* 76 genotypes.

## Discussion

Treatment with ASAQ-FDC and AL were highly effective against *P. falciparum* malaria in this clinical trial. Recrudescences did not have a significantly lower concentration of lumefantrine or DAQ day 7 [9]. Due to the low number of treatment failures, statistical analyses of the recrudescence genotypes were not meaningful. The number of reinfections was found to be high in the AL arm (30.0 %) and very high in the ASAQ-FDC arm (43.0 %) by day 42. In the ASAQ-FDC arm selection of the mutant allele *pfmdr1* 1246Y and the haplotypes *pfmdr1* YYY and *pfmdr1* NYY in reinfections was demonstrated in Liberia, showing a similar selection as observed in Tanzania [1] and in Mali [2]. The baseline prevalence of *pfmdr1* 1246Y at the time of this trial 2008–2009 was observed to be higher than in other countries in West Africa [38 % including mixed genotype infections compared to 0–25 % in 19 studies (search: years 2000–2014, n < 40, from West Africa)] as reported in literature [10]. The high *pfmdr1* 1246Y baseline prevalence may lead to the very high number of reinfections in the ASAQ-FDC arm in this trial, compared to the AL arm. Since 1246Y was observed to be selected in ASAQ-FDC reinfections, a higher baseline prevalence of the genotype can result in a larger pool of *pfmdr1* 1246Y carrying parasites that can survive the residual DAQ levels and result in more reinfections. Selection of *pfmdr1* 1246Y was observed mainly in early reinfections, while after day 35 the prevalence of 1246Y in reinfection was similar to the 1246Y prevalence before treatment. These results indicate that the concentration of DAQ was high enough to provide a selection pressure up to day 35. After this time-point all genotypes were able to survive the residual drug level and cause reinfection and there was no longer a protective or selective effect of the drug. This idea needs to be further elaborated by studying further efficacy trials with ASAQ and AL in West Africa. The 1246Y genotype has also been associated with ASAQ-FDC treatment failures [4]. Despite the high prevalence of 1246Y, few treatment failures were observed in this study, probably due to the high efficacy of the artesunate compound in the combination. Artemisinin resistance is defined as delayed parasite clearance after treatment with an artemisinin or ACT [11]. In this study a low proportion of patients were parasite positive on day 3 [9], indicating high efficacy of the artesunate compound.

In the AL arm, the wild-type alleles *pfcrt* K76, *pfmdr1* N86 and the *pfmdr1* haplotype NFD were selected in reinfecting parasites, by the drug pressure of residual levels of lumefantrine. Parasites harbouring *pfmdr1* N86 was not observed to reinfect significantly earlier, as has been suggested in previous studies [4, 6]. However it was demonstrated that parasites carrying *pfmdr1* N86 could cause reinfection in patients with higher lumefantrine concentrations, than parasites carrying *pfmdr1* 86Y. Patients with low lumefantrine levels could be reinfected with both N86 and 86Y alleles, while parasites with *pfmdr1* N86 could withstand intermediate drug concentrations. At high lumefantrine concentrations both genotypes would be killed and no reinfection could occur. This idea is supported by the observation that patients with reinfections had lower lumefantrine levels [9]. This is in agreement with an important study suggesting that reinfecting parasites with *pfmdr1* N86 as well as the *pfmdr1* N86/184F/D1246 haplotype can withstand higher lumefantrine concentrations based on day 7 data [12]. Both individual drug levels and the day of reinfection are key determinants for selection of reinfections and needs to be taken into account if available. This is especially important for AL-treated patients, in which the total lumefantrine dose received can vary significantly [13]. In studies from East Africa *pfmdr1* 184F is often selected in reinfections after AL treatment, which was not observed in this study. This could be due to the high baseline prevalence of *pfmdr1* NYD haplotype in this and a study in Benin [3], where *pfmdr1* N86 could be the main driving force for lumefantrine resistance, independently of the *pfmdr1*184 genotype.

The longer elimination half-life of the partner drug is a double-edged sword since although it can provide some post-prophylactic protection of individual patients, selection of reinfections can result in a more resistant parasite population over time. In high transmission areas, in Tanzania and Uganda, consistent treatment with AL overtime has probably resulted in significant increases in the prevalence of genotypes associated with AL treatment failure and reinfection [14, 15]. However, temporal changes of ASAQ resistance markers have not been observed after consistent use of AS–AQ in Zanzibar [16].

## Conclusions

In this first study investigating *pfcrt* and *pfmdr1* polymorphisms after the implementation of ACT in Liberia, selection of molecular markers in AL and ASAQ-FDC reinfections and high *pfmdr1* 1246Y baseline prevalence was demonstrated. The observation that parasites carrying *pfmdr1* N86 can reinfect patients with higher lumefantrine concentrations highlights the importance of studying drug levels after AL treatment, as there could be large inter-individual dose variations. It is important to further investigate variables governing selection of reinfections in individual studies and as well as over time, as they can be responsible for the step from selection of a reinfection to full resistance and treatment failures. To evaluate and advise the current treatment policy it is essential to monitor temporal changes in molecular resistance markers of treatments used in Liberia in conjunction with conventional efficacy testing.

## References

[1] Holmgren G, Hamrin J, Svard J, Mårtensson A, Gil JP, Björkman A (2007). Selection of *pfmdr1* mutations after amodiaquine monotherapy and amodiaquine plus artemisinin combination therapy in East Africa. Infect Genet Evol.

[2] Djimdé AA, Fofana B, Sagara I, Sidibe B, Toure S, Dembele D (2008). Efficacy, safety, and selection of molecular markers of drug resistance by two ACTs in Mali. Am J Trop Med Hyg.

[3] Dahlström S, Aubouy A, Maïga-Ascofaré O, Faucher JF, Wakpo A, Ezinmègnon S (2014). *Plasmodium falciparum* polymorphisms associated with ex vivo drug susceptibility and clinical effectiveness of artemisinin-based combination therapies in Benin. Antimicrob Agents Chemother.

[4] Venkatesan M, Gadalla NB, Stepniewska K, Dahal P, Nsanzabana C, Moriera C (2014). Polymorphisms in *Plasmodium falciparum* chloroquine resistance transporter and multidrug resistance 1 genes: parasite risk factors that affect treatment outcomes for *P. falciparum* malaria after artemether–lumefantrine and artesunate–amodiaquine. Am J Trop Med Hyg.

[5] Dokomajilar C, Nsobya SL, Greenhouse B, Rosenthal PJ, Dorsey G (2006). Selection of *Plasmodium falciparum pfmdr1* alleles following therapy with artemether–lumefantrine in an area of Uganda where malaria is highly endemic. Antimicrob Agents Chemother.

[6] Sisowath C, Ferreira PE, Bustamante LY, Dahlström S, Mårtensson A, Björkman A (2007). The role of *pfmdr1* in *Plasmodium falciparum* tolerance to artemether–lumefantrine in Africa. Trop Med Int Health.

[7] Jovel IT, Ferreira PE, Veiga MI, Malmberg M, Mårtensson A, Kaneko A (2014). Single nucleotide polymorphisms in *Plasmodium falciparum* V type H(+) pyrophosphatase gene (*pfvp2*) and their associations with *pfcrt* and *pfmdr1* polymorphisms. Infect Genet Evol.

[8] Checchi F, Durand R, Balkan S, Vonhm BT, Kollie JZ, Biberson P (2002). High *Plasmodium falciparum* resistance to chloroquine and sulfadoxine–pyrimethamine in Harper, Liberia: results in vivo and analysis of point mutations. Trans R Soc Trop Med Hyg.

[9] Schramm B, Valeh P, Baudin E, Mazinda CS, Smith R, Pinoges L (2013). Efficacy of artesunate–amodiaquine and artemether–lumefantrine fixed-dose combinations for the treatment of uncomplicated *Plasmodium falciparum* malaria among children aged six to 59 months in Nimba County, Liberia: an open-label randomized non-inferiority trial. Malar J..

[10] Molecular Surveyor *pfmdr1* and *pfcrt*. WorldWide Antimalarial Resistance Network (WWARN). http://www.wwarn.org/tracking-resistance/molecular-surveyor-pfmdr1-pfcrt. Accessed 1 Feb 2015.

[11] WHO: Update on artemisinin and ACT resistance. Geneva: World Health Organization; April 2016.

[12] Malmberg M, Ferreira PE, Tarning J, Ursing J, Ngasala B, Björkman A (2013). *Plasmodium falciparum* drug resistance phenotype as assessed by patient antimalarial drug levels and its association with pfmdr1 polymorphisms. J Infect Dis.

[13] Worldwide Antimalarial Resistance Network (WWARN) AL Dose Impact Study Group (2015). The effect of dose on the antimalarial efficacy of artemether–lumefantrine: a systematic review and pooled analysis of individual patient data. Lancet Infect Dis.

[14] Malmberg M, Ngasala B, Ferreira PE, Larsson E, Jovel I, Hjalmarsson A (2013). Temporal trends of molecular markers associated with artemether–lumefantrine tolerance/resistance in Bagamoyo district, Tanzania. Malar J.

[15] Mbogo GW, Nankoberanyi S, Tukwasibwe S, Baliraine FN, Nsobya SL, Conrad MD (2014). Temporal changes in prevalence of molecular markers mediating antimalarial drug resistance in a high malaria transmission setting in Uganda. Am J Trop Med Hyg.

[16] Fröberg G, Jörnhagen L, Morris U, Shakely D, Msellem MI, Gil JP (2012). Decreased prevalence of *Plasmodium falciparum* resistance markers to amodiaquine despite its wide scale use as ACT partner drug in Zanzibar. Malar J.

